# Energetic and Dynamic: How Mitochondria Meet Neuronal Energy Demands

**DOI:** 10.1371/journal.pbio.1001755

**Published:** 2013-12-31

**Authors:** Dzhamilja Safiulina, Allen Kaasik

**Affiliations:** Institute of Biomedicine and Translational Medicine, University of Tartu, Tartu, Estonia

## Abstract

Mitochondria are the power houses of the cell, but unlike the static structures portrayed in textbooks, they are dynamic organelles that move about the cell to deliver energy to locations in need. These organelles fuse with each other then split apart; some appear anchored and others more free to move around, and when damaged they are engulfed by autophagosomes. Together, these processes—mitochondrial trafficking, fusion and fission, and mitophagy—are best described by the term “mitochondrial dynamics”. The molecular machineries behind these events are relatively well known yet the precise dynamics in neurons remains under debate. Neurons pose a peculiar logistical challenge to mitochondria; how do these energy suppliers manage to traffic down long axons to deliver the requisite energy supply to distant parts of the cell? To date, the majority of neuronal mitochondrial dynamics studies have used cultured neurons, *Drosophila* larvae, zebrafish embryos, with occasional experiments in resting mouse nerves. However, a new study in this issue of *PLOS Biology* from Marija Sajic and colleagues provides an *in vivo* look at mitochondrial dynamics along resting and electrically active neurons of live anaesthetized mice.

## Introduction

All cells require energy, and thus mitochondrial dynamics are relevant to every cell type in the body; however, in long stretched out neurons the challenge presented to mitochondria is arguably much greater. Neurons are highly compartmentalized over long distances—as an example, the length of a human motoneuron could exceed one metre—and the energy requirements and the localization of those requirements are likely to vary in a pronounced and unpredictable manner. To meet these needs, mitochondria need machinery and mechanisms that sense energy requirements and enable them to respond accordingly. This evidence has come from *in vitro* models and also from *Drosophila*, worm, or *ex vivo* frog nerves, providing insights into activity dependent regulation of neuronal mitochondrial trafficking [1],[2]. However, there are limitations to how well cellular models, organotypic cultures, or excised nerves reflect the goings on in adult mammalian axons. Despite the earlier reports that imaged axonal transport of mitochondria *in vivo* [3]–[6], there are no studies that focus on neuronal activity-dependent mitochondrial motility in living adult mammals. Marija Sajic and colleagues now provide a first look at mitochondrial movement upon stimulation in a carefully exposed mouse saphenous nerve [7], affording us insight into the dynamics employed by these organelles to meet the energy challenges of neurons in response to varying levels of excitation.

## Mitochondrial Motility

**Figure 1:**
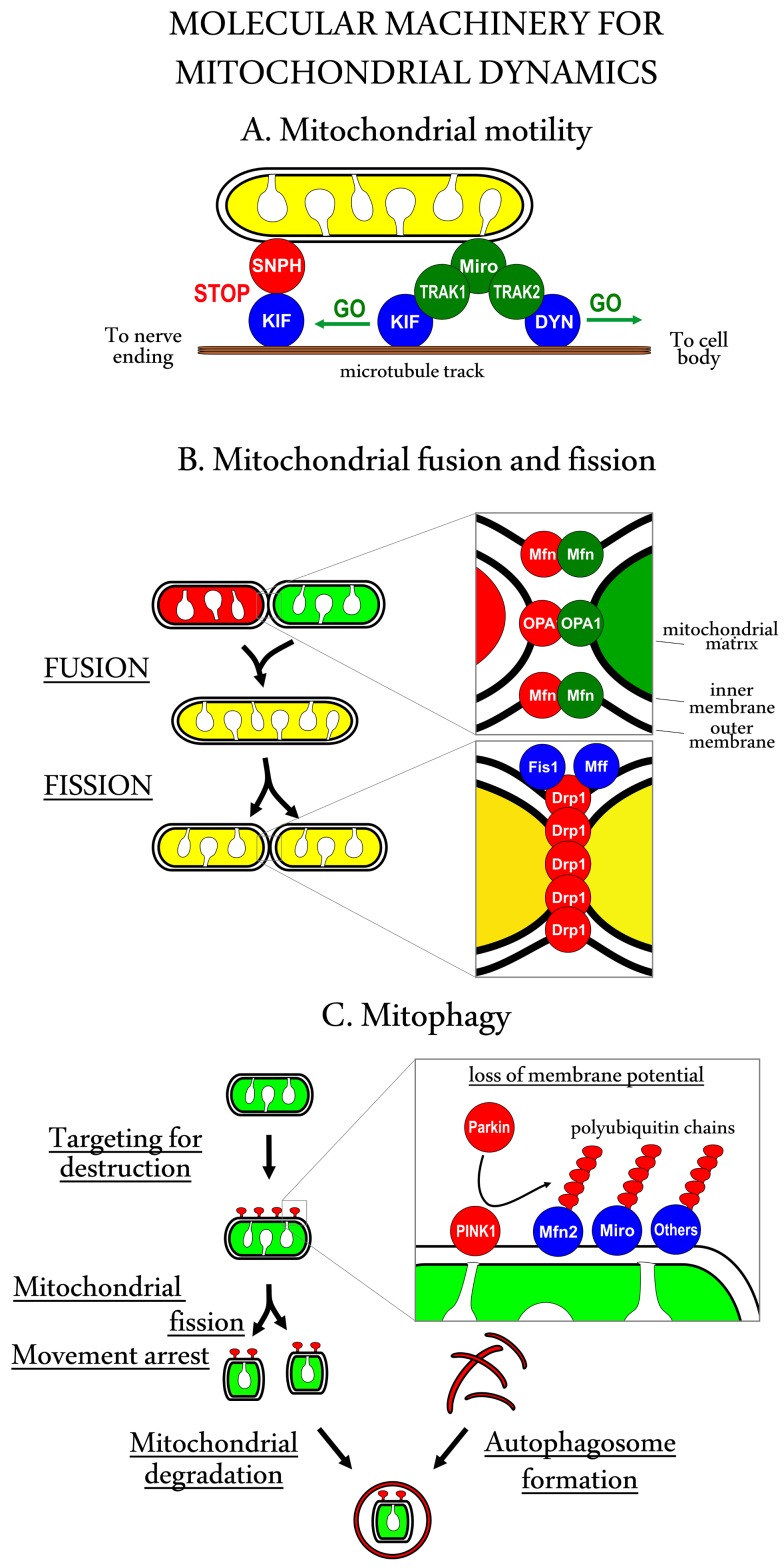
The molecular machinery of mitochondrial dynamics. (A) Mitochondrial transport is driven by the molecular motor proteins kinesin (KIF, for anterograde transport towards the nerve endings) and dynein (DYN, for retrograde transport towards the cell body) that move the cargo along microtubules. The adaptor proteins TRAK1 and TRAK2 link these motor proteins to Miro proteins located in the mitochondrial outer membrane. In addition, anchor proteins, such as Syntaphilin (SNPH), can dock the mitochondria in areas of high-energy requirements. (B) The mitochondrial outer membrane proteins Mitofusin (Mfn) 1 and 2 may link two mitochondria together and initiate fusion of their outer membranes. The outer membrane proteins Fis1 and/or Mff recruit cytosolic Drp1, which then mediates constriction and scission of the mitochondrion. (C) Mitochondrial depolarization leads to the accumulation of PINK1 on the outer membrane, which recruits the ubiquitin ligase Parkin to mitochondria. Parkin ubiquitinates mitochondrial outer membrane proteins (including Mfn2 to stop fusion and probably Miro1 to stop movement) and marks these mitochondria for degradation by autophagosomes.

The complexity of neuronal architecture requires highly organized organelle transport. Mitochondria move along microtubules, neurofilaments, and actin tracks, microtubule-based motility being the most important in axons (Figure 1). Considering the length of axons (an axon in the human sciatic nerve is approximately 1 m long) and the speed of mitochondrial movement (the mean velocity is approximately 0.7 µm/s, with a maximum of 3.4 µm/s), a mitochondrion can take several weeks to travel from the neuronal body to the synaptic terminal [8].

Mitochondrial transport is driven by the molecular motor proteins that use energy from ATP hydrolysis; kinesins carry organelles anterogradely towards the nerve endings, whereas dynein propels them retrogradely back towards the cell body. Mitochondria are transported by two kinesins, KIF1B and KIF5B, and mutations in the former are associated with Charcot-Marie-Tooth hereditary neuropathy type 2A1 [9], a peripheral nervous system disorder characterized by a progressive loss of touch sensation and muscle tissue across various body parts. The dynein shares some similarities with kinesin but functions as a multimeric complex, requiring dynactin for its interaction with microtubules. In addition, actin-based myosin motors are implicated in short-distance mitochondrial movements (e.g., within dendrites) and a recent study showed that myosins oppose microtubule-based axonal transport of mitochondria [10].

Several adaptor and anchor proteins are required to link mitochondria to the motors, e.g., mitochondrial Rho (Miro) GTPases [11]. Miro proteins bind to the trafficking kinesin protein (TRAK) family adaptor proteins, TRAK1 and TRAK2, which link mitochondria to microtubule-based motors [12]. Miro1 protein also acts as calcium-sensitive regulator of mitochondrial motility—when cytoplasmic Ca^2+^ levels increase, Miro1 arrests mitochondrial movement [13]–[15].

Last but not least, docking proteins or static anchors are also involved. Mitochondrial motility is a combination of saltatory and bidirectional movements and stationary docking. In neurons, approximately one-third of axonal mitochondria are highly motile whereas two-thirds are stationary. There is growing evidence that mitochondria are anchored by docking proteins at specific locations with high energy requirements.

## Fusion and Fission Dynamics

The molecular machineries behind mitochondrial fusion and fission events are relatively well known (Figure 1; reviewed in [16]). Mutations in the molecules responsible for fusion, Mitofusin-2 and OPA1, are related to the two neurological disorders Charcot-Marie-Tooth neuropathy, type 2A2 [17], and autosomal dominant optic atrophy, respectively [18],[19]. The regulation of fusion and fission events beyond the molecular machinery involved is less clear; fusion and fission are not random occurrences but form a cycle whereby fission typically follows fusion. Mitochondrial fission machinery may somehow sense mitochondrial length and become active when the mitochondrion is oversized and cease when mitochondria are smaller. In contrast, mitochondrial fusion events depend heavily on mitochondrial trafficking. Fusion only takes place when two mitochondria meet and motile mitochondria will be more likely to encounter one another. In cultured cortical neurons, only one in every 14th contact between mitochondria results in fusion. The frequency of fusion and fission events in neuronal axons is rather low compared with cell lines and also varies between different subtypes of neurons (0.023±0.003 fusions/mitochondrion/min and 0.023±0.003 fissions/mitochondrion/min in cultured mixed cortical neurons and 0.045±0.006 fusions/mitochondrion/min and 0.039±0.005 fissions/mitochondrion/min in cultured cerebellar granule neurons) [20].

What is the exact physiological purpose of mitochondrial fusion and fission? On one hand, frequent fusion and fission events serve to maintain homogeneity within the mitochondrial population in the cell, thereby preserving its well-being. Damaged mitochondria will fuse with healthy ones, diluting the damage and sharing components of “healthy” mitochondria. On the other hand, it has been suggested that fission could shuttle off mutated copies of the mitochondrial genome into “garbage” mitochondria that are then destroyed by the autophagosome [21]. If so, then the fusion-fission cycle will, together with mitophagy, help improve the health of the mitochondrial population.

Another purpose of fusion and fission is to exert control over mitochondrial length. Under steady state conditions, the number of fusion and fission events is perfectly balanced, thus keeping mitochondrial length stable. However, when the fusion machinery is inhibited or fission machinery is activated we see mitochondrial shortening. A common misinterpretation is that shorter mitochondria necessarily suggest activated fission; clearly this phenotype could equally be due to inhibited fusion [20].

Although mitochondrial length has been considered important for cellular physiology, precisely why it is important has remained unclear. Longer or shorter mitochondria may differ in function (ATP production, reactive oxygen species generation) or in sensitivity to apoptotic stimuli or autophagic removal (reviewed in [22]). However, activation of mitochondrial fission (or inhibition of fusion) will lead not only to shorter/smaller mitochondria but will also increase the number of mitochondria per cell. More (smaller) mitochondria allow more precise fine-tuning of their distribution, whereas fewer (larger) mitochondria are more coarsely (less evenly) distributable, which may result in an energy deficit in some compartments.

## Relevance of Mitochondrial Dynamics *In Vivo*

**Figure 2:**
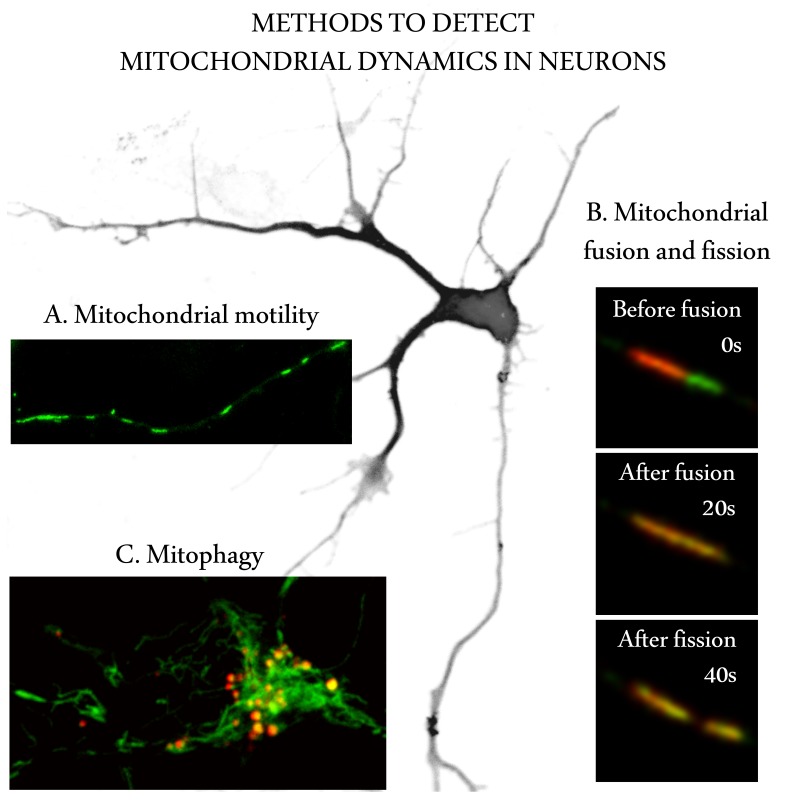
Image-based techniques to quantify mitochondrial dynamics in neurons. (A) Mitochondrial motility can be visualized using time-lapse microscopy in neurons that are either transfected with plasmids expressing mitochondrially targeted fluorescent proteins or loaded with mitochondrion-specific fluorescent dyes (Video S1). Mitochondria are tracked in the image series either manually or automatically using specific software packages. Various movement parameters (e.g., number and percentage of mobile and stationary mitochondria, the number, length and direction of runs or tracks the mitochondria make, their maximum and average velocity) can be calculated from the data obtained. (B) Mitochondrial fusion and fission can be measured using photoactivatable or photoconvertable mitochondrially targeted fluorescent proteins such as Kikume [20],[23]. The middle panel shows a fusion event between green- and red-emitting mitochondria, yielding a yellow fusion product after the contents of the mitochondrial matrices are mixed (Video S2). The lower panel demonstrates that the fusion was followed by a fission event. The fate of photoactivated mitochondria can be followed throughout the time-lapse series and the fusion and fission rates calculated. (C) Detection of mitophagy in the cell body of a primary cortical neuron expressing a mitochondrially targeted pH-dependent protein Keima [24],[25] whose excitation spectrum shifts from 440 nm to 586 nm (green to red in photo) when mitochondria are delivered to acidic lysosomes, thus enabling easy quantification of mitophagy. An alternative approach is to quantify the co-localization of an autophagosome marker (EGFP-LC3) with a mitochondrial marker.

Most studies of mitochondrial dynamics rely on cultured cells and neurons, where mitochondria can be visualized using mitochondria using mitochondria-targeted fluorescent proteins or dyes (Figure 2; Videos S1 and S2). Mitochondrial dynamics have also been studied in invertebrates, particularly in *Drosophila* larval motor axons, but we have very limited information on mitochondrial dynamics in mammalian neuronal tissues.

In this issue of *PLOS Biology*, Sajic et al. [7] present a study analyzing mitochondrial trafficking in axons imaged in the living mouse. The study demonstrates that both electrical and pharmacological stimulation of the saphenous nerve (a branch of the femoral nerve) increases mitochondrial trafficking in axons. Interestingly, stationary mitochondria become shorter suggesting that the fusion/fission process is used to produce more mobile mitochondria. Surprisingly, these mobile mitochondria did not accumulate near Nodes of Ranvier, but travelled in an anterograde direction toward peripheral nerve terminals. It was generally believed that mitochondria are usually present in areas of high energy demand, such as in synaptic terminals where ATP is required to mobilize vesicles for neurotransmission and in sites of action potential regeneration, such as Nodes of Ranvier. One might expect these sites to show the most fluctuations in energy demands and that the mitochondria would move in and out of these regions in response to physiological impulse activity. However, data presented by Sajic et al. suggest that the peripheral terminals of sensory axons, and not the Nodes of Ranvier, represent sites of particularly high metabolic demand during physiological high-frequency conduction. Together, these results suggest that mitochondrial trafficking and fusion fission dynamics are tightly orchestrated *in vivo* to meet the fluctuating and compartmentalized energy requirements of the neuron. Indeed, this study is one of the first to demonstrate the relevance of mitochondrial dynamics *in vivo*.

## Supporting Information

Video S1 **Mitochondrial trafficking in a cortical neuron expressing mitochondrially targeted fluorescent protein.**
(MPG)

Video S2 **Fusion of green- and red-emitting mitochondria within a cortical neuron expressing mitochondrially targeted photoconvertable Kikume green-red fluorescent protein.**
(AVI)

## Abbreviations

Miro: mitochondrial Rho GTPase
Mfn: mitofusin
TRAK: trafficking kinesin protein
